# On the lethal mechanism of class III ferroptosis inducers

**DOI:** 10.70401/fos.2026.0028

**Published:** 2026-05-20

**Authors:** Alby Joseph, Scott J. Dixon

**Affiliations:** Department of Biology, Stanford University, Stanford, CA 94305, USA.

**Keywords:** Lipid peroxidation, necrosis, erastin, RSL3, FIN56, CIL56, tegavivint

## Abstract

Ferroptosis is an oxidative form of non-apoptotic cell death that is important for human biology. This process can be induced in cultured cells by at least four structurally and mechanistically distinct classes of ferroptosis inducing (FIN) small molecules. These four classes of FINs are distinguished based on molecular target and mechanism of action. The lethal mechanism of the prototypic oxime-containing class III FIN, FIN56, is unique and poorly understood. FIN56 is proposed to cause ferroptosis by depleting coenzyme Q10 and degrading glutathione peroxidase 4 (GPX4). Curiously, the FIN56 analogs caspase independent lethal 56 (CIL56) and tegavivint also trigger non-apoptotic cell death but not ferroptosis. Tegavivint is a drug candidate currently being tested in humans for the treatment of cancer. Here, we review our understanding of the FIN56 lethal mechanism with a view to guiding future investigations into a privileged chemical scaffold that possesses unusual lethal activity in cancer cells.

## 1. Introduction

Ferroptosis is a non-apoptotic form of cell death driven by the lethal accumulation of membrane lipid peroxides^[1,2]^. Ferroptosis has been implicated in several pathological contexts, including tumorigenesis, neurodegeneration, autoimmunity, and organ injury^[3]^. It may be possible to target ferroptosis therapeutically, which is attractive in the context of cancer and other diseases^[4]^. Under homeostatic conditions, polyunsaturated fatty acid (PUFA)-containing membrane phospholipids are regularly peroxidized^[5]^. PUFA peroxidation can be catalyzed enzymatically by iron-dependent enzymes or occur spontaneously through the reaction of labile intracellular iron with hydroperoxides to generate hydroperoxyl radicals^[6,7]^. Unfettered lipid radical propagation on multiple membranes of the cell occurs during the execution of ferroptosis^[2]^. Excessive plasma membrane lipid peroxidation increases membrane tension and leads to membrane rupture^[8]^. Multiple cell-intrinsic enzymes and metabolites function to prevent membrane lipid peroxidation and ferroptosis, most notably glutathione peroxidase 4 (GPX4)^[9]^.

Ferroptosis was initially characterized using lethal small molecules. These lethal molecules emerged from screens for compounds that selectively killed engineered cancer cells harboring oncogenic mutant RAS^[10,11]^. Despite this early and obvious connection between ferroptosis-inducing small molecules and cancer therapy, there is to date no clinical translation of ferroptosis-inducing small molecules. One reason is that we lack suitable drug candidates to take into humans^[12]^. For example, covalent GPX4 inhibitors are limited by high levels of on-target toxicity in normal tissues^[13–15]^. It is therefore of interest to explore agents that could inhibit GPX4 in a different manner, or perhaps engage alternative targets altogether. Here, we briefly review the canonical chemical inducers of ferroptosis. We then focus on FIN56, the class III ferroptosis inducer (FIN) that has unique properties and potential utility compared to other classes of FINs.

## 2. Ferroptosis-Inducing Compounds

Four distinct classes of FINs have been described (Figure 1A,B,C,D). The first two FIN classes were identified in early studies. Class I FINs (e.g., erastin, sulfasalazine and glutamate) inhibit cystine import via the system X_c_^−^ cystine/glutamate antiporter (Figure 1A)^[16]^. Cystine deprivation prevents the de novo synthesis of reduced glutathione (GSH) and other cysteine thiol-dependent metabolites that defend against lipid peroxidation^[16–19]^. GPX4 uses GSH as a co-substrate to enzymatically reduce potentially toxic lipid hydroperoxides (L-OOH) to non-toxic lipid alcohols (L-OH)^[20]^. Therefore, class I FINs inactivate GPX4 indirectly. Class II FINs, including (1*S*, 3*R*)-RSL3 (hereafter, RSL3), ML162, ML210, JKE-1674, and Compound 28, directly inhibit GPX4 by forming a covalent adduct with the active site selenocysteine (Figure 1B)^[11,14,17,21,22]^ Thus, class I and class II FINs cause ferroptosis by disrupting the GSH and GPX4-dependent cellular mechanism that suppresses the accumulation of membrane lipid peroxides.

The class I and class II FINs have been thoroughly investigated in terms of targets and lethal mechanisms of action. Two other classes of ferroptosis inducing molecules (III and IV) reported in the literature have received less attention. The 1,2-dioxolane FINO2 is categorized as a class IV FIN (Figure 1D)^[23,24]^. The proposed FINO2 lethal mechanism involves direct reaction with free ferrous iron at the endoperoxide functional group^[24,25]^. This reaction between FINO2 and iron may generate free radicals that can propagate to polyunsaturated lipid species and trigger ferroptosis^[26]^. FINO2 also appears to indirectly inactivate GPX4 in cell lysate-based assays^[25]^. FINO2 does not deplete GSH and NMR-based assays confirmed that FINO2 is neither an active site ligand nor allosteric ligand of GPX4^[25]^. The free radical-generating endoperoxide is therefore best understood as the mechanism by which FINO2 induces ferroptosis. The endoperoxide-containing antimalarial artemisinin and its derivatives may also promote ferroptosis, though less specifically, by iron-dependent radical generation, and could fit the definition of class IV FINs^[27–29]^.

The structurally distinct oxime-containing compound FIN56 is the prototypic class III FIN (Figure 1C). The proposed FIN56 lethal mechanism is incompletely understood. As described below, FIN56 appears to cause ferroptosis by inhibiting GPX4 activity^[23,25]^. Further resolving the FIN56 lethal mechanism of action is of interest for two reasons. First, FIN56 may inhibit GPX4 through a non-covalent mechanism, a unique mode of action compared to class II FINs. Understanding this mechanism may illuminate novel principles of post-translational GPX4 regulation which could potentially be more safely exploited than covalent GPX4 inhibitors in humans. Second, a structural analog of FIN56 that induces a distinct form of non-apoptotic cell death has advanced into human clinical trials for the treatment of cancer, opening a new window into the potential utility of FIN56 or derivatives thereof in the clinical setting. Overall, the oxime-containing FIN56 chemical structure appears to be a privileged scaffold for the induction of non-apoptotic cell death.

## 3. The FIN56 Lethal Mechanism of Action

FIN56 was not directly identified from the same oncogenic RAS selective lethal chemical screens that yielded erastin and RSL3. Rather, the starting point for FIN56 was a screen for lethal molecules that caused cell death without evidence of pro-apoptotic caspase 3 and caspase 7 activation^[23]^. One hit from the screen was named caspase-independent lethal 56 (CIL56, Figure 1E). Phenotypically, CIL56 appeared to trigger a mixture of ferroptosis and some other lethal mechanism. This motivated a synthetic chemistry effort to isolate the ferroptosis-inducing activity of the CIL56 scaffold. This synthetic campaign resulted in identification of FIN56 as a ‘pure’ ferroptosis inducer with sub-micromolar potency^[23]^.

Consistent with other FINs, FIN56 lethal activity is suppressed by the iron chelator deferoxamine and the lipophilic radical trapping antioxidant α-tocopherol^[23,30]^. FIN56 activity is also suppressed by supplementing cells with the reducing agent beta-mercaptoethanol to increase GSH synthesis^[25]^. However, unlike class I FINs, FIN56 does not deplete GSH^[23]^. There is also no evidence that FIN56 directly binds or forms a covalent adduct with GPX4. Indeed, the FIN56 structure does not contain an obvious electrophile like class II FINs (Figure 1C). There is also no evidence that FIN56 directly generates radical species or directly oxidizes ferrous iron, like the class IV FIN, FINO2^[25]^. However, FIN56 treatment inhibits GPX4 activity detected in cell lysates and reduces GPX4 protein abundance over time without changing *GPX4* mRNA levels^[23,25]^. These observations suggest a model whereby FIN56 triggers ferroptosis by decreasing GPX4 protein abundance post-translationally.

To identify the target of FIN56, a FIN56 affinity probe was used to enrich for direct protein binders from cell lysates. The top candidate to emerge from this analysis, following short hairpin RNA (shRNA) functional validation, was squalene synthase (SQS, encoded by *FDFT1*)^[23]^. FIN56 is proposed to activate SQS, since shRNA-mediated knockdown of *FDFT1* or chemical inhibition of SQS rescues cells from FIN56-induced cell death^[23]^. SQS catalyzes the dimerization of farnesyl pyrophosphate (FPP), a branchpoint intermediate of the mevalonate pathway, to produce squalene (Figure 2A). While FPP can be funneled to several metabolic pathways, SQS commits FPP to cholesterol biosynthesis (Figure 2A). A potential mechanistic model is that FIN56 activates SQS, leading to increased metabolic flux of FPP to sterol biosynthesis with several consequences.

First, upstream of FPP, SQS activation by FIN56 may reduce the levels of isopentenyl pyrophosphate (IPP). Isopentenylation via IPP is a post-transcriptional modification that stabilizes the selenocysteine-specific tRNA required for translation of GPX4^[31]^. Lower levels of IPP due to accelerated flux through SQS could decrease GPX4 protein synthesis and explain FIN56-induced GPX4 protein depletion. Consistent with this model, supplementation with mevalonate partially suppresses the lethality of FIN56^[23]^. Co-treatment with statins, which inhibit mevalonate synthesis, also sensitize cells to FIN56^[23]^. However, genetic knockdown of tRNA isopentenyltransferase 1 (*TRIT1*), which catalyzes the selenocysteine-specific tRNA isopentenylation does not reduce GPX4 protein levels^[23]^, potentially calling the isopentenylation model into question.

Second, SQS activation by FIN56 may increase metabolic flux into cholesterol synthesis at the expense of a different FPP-derived metabolite, ubiquinone (Coenzyme Q10, CoQ_10_). The reduced form of CoQ_10_, ubiquinol, is an important ferroptosis-suppressing metabolite synthesized by the oxidoreductase ferroptosis suppressor protein 1 (FSP1)^[32,33]^. Ubiquinol acts as a lipid-soluble radical trapping antioxidant to terminate lipid peroxidation. Supplementation with FPP or idebenone (a synthetic analog of CoQ_10_) protects cells from FIN56 lethality, with idebenone likely functioning directly as a radical trapping agent^[23]^. Of note, these compounds do not restore GPX4 protein levels upon FIN56 treatment^[23]^. Although levels of ubiquinone or ubiquinol in FIN56-treated cells have not been directly measured, there is indirect evidence that FIN56 indeed modulates CoQ_10_ in parallel to its effect on GPX4. Lung cancer cell lines in which kelch-like ECH-associated protein 1 (KEAP1) is inactivated are resistant to class I and class II FINs because transcription of FSP1 is upregulated. By contrast, KEAP1 deficiency does not affect sensitivity to FIN56, presumably because this agent uniquely depletes CoQ_10_ which counteracts the otherwise protective effect of FSP1 upregulation^[34]^. Thus, the induction of ferroptosis by FIN56 may require the combined effect of GPX4 protein loss and CoQ_10_ depletion. Indeed, loss of CoQ_10_ regeneration by FSP1 alone is generally insufficient to trigger ferroptosis in most cancer cells so long as GPX4 activity remains intact^[32,35,36]^.

Third, the depletion of FPP-derived metabolites other than CoQ_10_, or an increase in cholesterol biosynthetic pathway intermediates, including squalene itself, could also contribute to the FIN56 lethal mechanism (Figure 2A). However, this is highly speculative, and if anything, it now appears that activation of the cholesterol biosynthesis pathway could protect against ferroptosis: two products of this pathway, squalene and 7-dehydrocholesterol (7-DHC), can both inhibit ferroptosis in some cells^[37–39]^.

## 4. Unresolved Mechanistic Questions

While FIN56-mediated SQS activation provides one possible explanation for ferroptosis induction, much about this model remains unclear. For example, how SQS activation would cause GPX4 protein to be depleted still remains to be elucidated. An alternative model proposes that FIN56 treatment leads to the destruction of GPX4 by upregulating macroautophagy (referred to as autophagy)^[40]^. In support of this model, FIN56, but not other FINs, causes the rapid formation of autolysosomes which is not suppressed by the radical trapping antioxidant, ferrostatin-1 (Fer-1), implying that it is an event that occurs upstream of lipid peroxidation^[41]^. Chemical and genetic inhibition of autophagy attenuates the reduction in GPX4 protein levels caused by FIN56^[40]^. In addition, inhibition of mammalian target of rapamycin (mTOR), which promotes autophagy, synergizes with FIN56 to cause cell death^[40]^. Whether FIN56 might induce autophagy by engaging SQS or through another mechanism is not clear. Moreover, how the induction of autophagy would result in selective GPX4 degradation is unknown, and a general upregulation of autophagy might be expected to have effects on lipids, iron metabolism, and other effectors of ferroptosis beyond GPX4. Another type of autophagy, chaperone-mediated autophagy (CMA), promotes the degradation of GPX4 in response to class I FIN erastin[42]. However, FIN56 does not engage CMA^[40]^. There also exists a different line of evidence that FIN56 promotes lysosomal membrane permeabilization in glioma cell lines^[43]^. This effect would seem to counter autophagy-dependent GPX4 degradation and may be specific to the cell type. Thus, it is unclear whether upregulation of autophagy offers a compelling explanation for how FIN56 might degrade GPX4 and trigger ferroptosis.

Overall, deeper scrutiny of whether FIN56-mediated GPX4 protein depletion is sufficient to drive ferroptosis is likely needed. The initial observation that FIN56 treatment leads to GPX4 protein depletion was made in cells co-treated with a ferroptosis inhibitor for 10 hours^[23,25]^. It would be useful to measure GPX4 protein abundance before the onset of cell death and in the absence of a ferroptosis inhibitor. The key question is whether FIN56 reduces GPX4 protein levels fast enough and low enough to induce ferroptosis, or whether ferroptosis is initiated by FIN56 before any noticeable loss of GPX4 protein.

There is also reason to reexamine whether SQS is likely to be the most relevant biochemical target of FIN56 in the first place. In the original study, FIN56 was functionalized at the oxime to develop the probe used for resin immobilization and subsequent mass spectrometry-based proteomics^[23]^. However, FIN56 structure-activity relationship (SAR) analysis indicates that the ketoxime functional group (generally referred to as oxime herein) of FIN56 is essential for lethal activity and that the synthetic ketone precursor is inactive^[23]^. The FIN56 chemoproteomic probe destroys this essential oxime group. It is therefore possible to imagine in retrospect that this chemoproteomic probe might struggle to interact with the protein target(s) most relevant for the induction of ferroptosis without the oxime in place. A distinct and simple model to explain the ability of FIN56 to induce ferroptosis is that this molecule binds and inhibits GPX4 directly, but allosterically and non-covalently. Direct FIN56-GPX4 interaction would be consistent with much of the available phenotypic evidence and potentially also explain how GPX4 protein could be lost over time if FIN56 binding also disrupts protein stability. While GPX4 did not emerge as a hit in the FIN56 chemoproteomic study that identified SQS, this may be because the affinity probe disrupted on-target binding for the reasons described above.

## 5. CIL56: The Odd Cousin of FIN56

An interesting and unexplained aspect of the FIN56 lethal mechanism relates to its connection with lipid metabolism. 5-(tetradeclyoxy)-2-furoic acid (TOFA), a chemical inhibitor of acetyl-CoA carboxylase (ACC) enzymes, blocks GPX4 depletion following FIN56 treatment and inhibits FIN56-induced cell death^[18]^. The potential mechanism linking ACC activity, GPX4 protein abundance, and FIN56 activity is an open question. TOFA is also used as a tool to inhibit the lethal activity of CIL56^[44–46]^. While there is structural and mechanistic overlap between FIN56 and CIL56, the two molecules ultimately seem to engage distinct lethal mechanisms.

FIN56 was synthesized as a derivative of CIL56 (Figure 1E) ^[23]^. The two lethal compounds share the same tricyclic central 9H-fluoren-9-one oxime core and differ in their sulfonamide pendant groups (Figure 1C,E and Table 1). SAR studies show that, like FIN56, the oxime moiety of CIL56 is essential for lethal activity^[45]^. Despite the obvious structural similarity between CIL56 and FIN56, iron chelators and lipophilic antioxidants only partially inhibit the lethality of CIL56 in BJ-eLR fibroblast cells^[23,30]^ and do not inhibit CIL56-induced cell death at all in many other cancer cell lines^[44,45]^. Small molecules predicted to inhibit prenylation-dependent membrane targeting and cytochrome P450 enzymes involved in hormone metabolism also uniquely suppress the non-ferroptotic lethality of CIL56, but not that of FIN56[30]. These findings tend to suggest that CIL56 is capable of triggering a distinct lethal mechanism. Indeed, multiple lines of evidence show that CIL56 can induce a form of cell death that is distinct from ferroptosis and other regulated forms of non-apoptotic cell death, termed lipid-dependent necrosis (LiDN) ^[30,45]^. Another structurally-related, oxime-containing small molecule, tegavivint (Figure 1E and Table 1), is likewise able to induce LiDN in multiple cancer cell lines (Figure 2B)^[46]^. Efforts to characterize the LiDN mechanism are summarized below.

Chemical genetics screens using CIL56 in three different cancer cell lines identified lipid metabolism and protein *S*-palmitoylation genes as key effectors of CIL56 activity^[44]^. Top suppressor genes include the rate-limiting de novo lipogenesis enzyme acetyl-CoA carboxylase 1 (ACC1, encoded by *ACACA*), the palmitoyltransferase zDHHC palmitoyltransferase 5 (*ZDHHC5*) and the reductase trans-2,3-enoyl-CoA reductase (*TECR*), which is involved in fatty acid synthesis^[44]^. Genetic knockout or knockdown of *ACACA*, *ZDHHC5* or *TECR* is sufficient to inhibit CIL56-induced LiDN, while *TECR* is clearly essential for tegavivint to induce LiDN^[44–46]^. The discovery that *ACACA* promoted CIL56 lethality was the initial motivation to use TOFA as a chemical inhibitor of CIL56-induced cell death. Though TOFA also inhibits FIN56 lethality, whether genetic disruption of *ACACA* is sufficient to inhibit FIN56-induced ferroptosis has not been tested.

The 16-carbon saturated fatty acid palmitate (C16:0) promotes both CIL56- and tegavivint-induced LiDN. Palmitate can be synthesized de novo in an ACC1-dependent manner that also requires the enzyme fatty acid synthase (FASN). Independent of ACC1 and FASN, TECR can also catalyze the synthesis of palmitoyl-CoA as the terminal step in a sphingolipid catabolic pathway^[47]^. It is this specific activity of TECR that appears to promote LiDN following CIL56 or tegavivint treatment[46].

ZDHHC enzymes catalyze S-palmitoylation, a reversible post-translational modification in which palmitate is appended to cysteine residues via a thioester linkage. Like TOFA, the S-palmitoylation inhibitor 2-bromopalmitate (2-BP) potently inhibits LiDN in response to CIL56 and tegavivint (Figure 2B). Based on genetic knockout studies and large-scale correlation analysis of cell line sensitivity with basal gene expression, *ZDHHC20*, rather than *ZDHHC5*, may be required for tegavivint to promote LiDN[46]. Of note, both ZDHHC20 and ZDHHC5 are reported to localize predominantly to the plasma membrane rather than internal compartments, unlike most ZDHHC family acyltransferases^[48]^. Taken together, lipid metabolism and plasma membrane protein palmitoylation seem critical for the execution of LiDN (Figure 2B). Whether this is also true for FIN56 is unclear. The highly conserved nature of the chemical structures of these oxime-containing lethal compounds suggests there may be a shared molecular basis for activity. Yet, FIN56 induces ferroptosis whereas CIL56 and tegavivint induce LiDN in most cells, a distinct non-ferroptotic mechanism. A model that could explain these observations has yet to be presented.

## 6. Oxime Mechanisms of Action

The oxime functional group of FIN56, CIL56, and tegavivint may give rise to unique chemistry inside the cell that triggers non-apoptotic cell death, at least in immortalized or cancerous cell lines. Oximes and oxime-derived functional groups appear in synthetic chemistry methodologies, plant-derived natural products, and biologically active synthetic compounds, including several FDA-approved drugs^[49,50]^. A notable example is pralidoxime, an FDA-approved drug used to treat organophosphate poisoning. Pralidoxime has a reactivity-based mechanism. The oxime acts as a nucleophile to cleave the phosphate ester bond of the inactivated organophosphate-enzyme complex by nucleophilic substitution and regenerates the active enzyme. Another example is provided by the oxime derivatives of cephalosporin, which act as anti-microbial agents. In this case, the oxime appears to improve pharmacokinetic properties. Most commercially available oximes, including pralidoxime, are not lethal to cancer cells^[46]^. However, a series of oxime-containing indirubin derivatives can induce caspase-independent cancer cell death^[51–53]^. This was a surprising result as the parent molecule indirubin, a bis-indole natural product, has anti-proliferative, pro-apoptotic effects in cancer (particularly, leukemia) through cyclin-dependent kinase inhibition^[54]^. It is unclear how installation of an oxime on indirubin analogues improves cytotoxic activity and purportedly alters the mechanism of action. In addition to indirubin, there are reports that the installation of an oxime onto other natural products improves lethal or anti-proliferative activity^[55,56]^.

## 7. Clinical Translation of Oxime-Containing Inducers of Non-Apoptotic Cell Death

Tegavivint (also called tegatrabetan, BC-2059) is a drug candidate that has advanced into phase I/II human clinical trials for several cancer indications. Promising safety and tolerability profiles are reported for tegavivint in preliminary studies, but definitive analyses remain outstandinh^[57,58]^. Tegavivint was first developed as a more potent and drug-like analog of CIL56 with a 2,7-sulfonamide disubstituted 9,10-anthraquinone core and two oxime moieties instead of one (Figure 1E and Table 1)^[59]^. Tegavivint can inhibit cancer cell growth in HT-29 colon cancer cells and other cell lines, both in vitro and *in vivo*^[59]^. Tegavivint was initially suggested to induce apoptosis by destabilizing β-catenin^[59,60]^. More specifically, tegavivint is proposed to target transducin beta-like protein 1 (TBL1), inhibiting its interaction with β-catenin^[60]^. The model is that disruption of the TBL1:β-catenin complex leads to enhanced degradation of β-catenin, inhibiting oncogenic Wnt-dependent transcriptional activity and ultimately causing cell death^[60]^. Biochemical assays including co-immunoprecipitation and cellular thermal shift assay support this model in part. However, profiling in the Broad Institute’s PRISM platform indicates that tegavivint is broadly lethal to over 800 human cancer cell lines without any notable selectivity towards cells with activating mutations in the Wnt/β-catenin pathway, suggesting the existence of an alternative mechanism, at least in cultured cells^[46]^.

In initial studies with tegavivint, “apoptotic” cell death was assessed by annexin V staining^[59,60]^, which cannot definitively establish that apoptosis has occurred. Indeed, in HT-1080 and other cancer cell lines, lethal doses of CIL56 and tegavivint do not increase the abundance of the apoptosis marker cleaved caspase 3 and cell death in response to these agents is not blocked by the pan-caspase inhibitor quinoline-Val-Asp-Difluorophenoxymethylketone (Q-VD-OPh), inconsistent with the induction of apoptosis^[46]^. Rather, results suggest that tegavivint can induce TECR-dependent LiDN, both in cultured cells and in mouse xenograft tumor models. It remains possible that in human tumors tegavivint induces apoptosis by inhibiting the Wnt pathway, but induction of LiDN may provide an alternative explanation for any positive clinical signals that are observed in the future.

## 8. Discussion and Concluding Thoughts

Almost fifteen years since the original description of ferroptosis in 2012 we may do well to ask: does it still make sense to group ferroptosis inducing compounds into four classes? Based on the evidence summarized here, we argue that it does, given the distinct structures, targets and mechanisms of class I-IV FINs. However, further expansion of the FIN concept is also needed to account for new molecules, targets and mechanisms. For example, the discovery of an orthogonal axis of ferroptosis suppression by FSP1 resulted in a mechanistically distinct class of ferroptosis inducers that includes ferroptosis sensitizer 1 (FSEN1) and FSP1 inhibitor iFSP1, among others (Figure 1F)^[29,61,62]^. The concept that GPX4 and FSP1 act in parallel axes to suppress ferroptosis suggests new ways to induce ferroptosis in cancer, with evidence that single-agent targeting of FSP1 may be useful in fully immune-competent mouse models of metastatic cancer^[63,64]^. Unlike Class II FINs, FIN56 may interfere non-covalently with both the GPX4 and the FSP1/CoQ_10_ mechanisms of ferroptosis suppression^[34]^. Because of this potentially unique dual mechanism of action, the concept of a unique “class III FIN201D still appears to hold value. However, as noted above, there remain critical questions concerning the FIN56 lethal mechanism, including the target of this agent and the role for GPX4 protein loss in ferroptosis induction. New approaches may be needed to identify additional unique features of FIN56 that truly distinguish its lethal mechanism from class II FINs.

With regards to the application of FIN56 therapeutically, there are several important open questions at the chemical, cellular and organismal levels. First, the best cancer indication for FIN56 is not clear; the sensitivity of different cancer types to FIN56 has not been thoroughly characterized, even in culture. Second, whether FIN56 may synergize with other FINs or other cytotoxic agents has not been studied. Third, the safety profile of FIN56 is poorly understood; whether FIN56 is toxic to non-cancerous or non-transformed cells in culture or *in vivo* has not been investigated in enough depth. Fourth, the *in vivo* anti-tumor activity of FIN56 is underexplored. A few studies have evaluated FIN56 in murine tumor models. For example, FIN56 as a monotherapy decreased tumor volume in a mouse xenograft model in which human glioblastoma LN-229 cells were implanted subcutaneously in nude mice^[43]^. However, details on FIN56 treatment, including dosing and administration, were not provided^[43]^. In another study, FIN56 was encapsulated in inhalable liposomal nanoparticles in combination with another small molecule proposed to increase reactive oxygen species and thereby promote ferroptosis^[65]^. Administration of FIN56 in this way inhibited tumor growth in an orthotopic murine model of lung adenocarcinoma using human A549 cells in nude mice^[65]^. The field would benefit from additional studies investigating the efficacy of FIN56 in tumor models and deeper characterization of the pharmacokinetics of this compound *in vivo*.

Given the known or potential challenges associated with system x_c_− inhibitors, GPX4 inhibitors, and FSP1 inhibitors^[66]^, there is a need for novel chemical matter and/or mechanistic targets to therapeutically activate ferroptosis in the oncology setting. One possibility is that FIN56, through a dual mechanism of action that could include non-covalent inhibition of GPX4, will be a less toxic alternative to class II FINs for the therapeutic induction of ferroptosis to treat cancer. But perhaps other non-apoptotic approaches beyond ferroptosis are also possible. Most notably, could tegavivint-induced LiDN be activated in tumors where tegavivint is currently being tested? In turn, could these ongoing clinical trials further motivate studies of FIN56 *in vivo*, with the view that these related oxime-containing molecules have privileged activities in animals? At a more fundamental level, how do CIL56 and tegavivint induce LiDN, and how is LiDN distinguished from ferroptosis? These important questions could lead both to new biological understandings and new advances for cancer therapy.

## Figures and Tables

**Figure 1.**
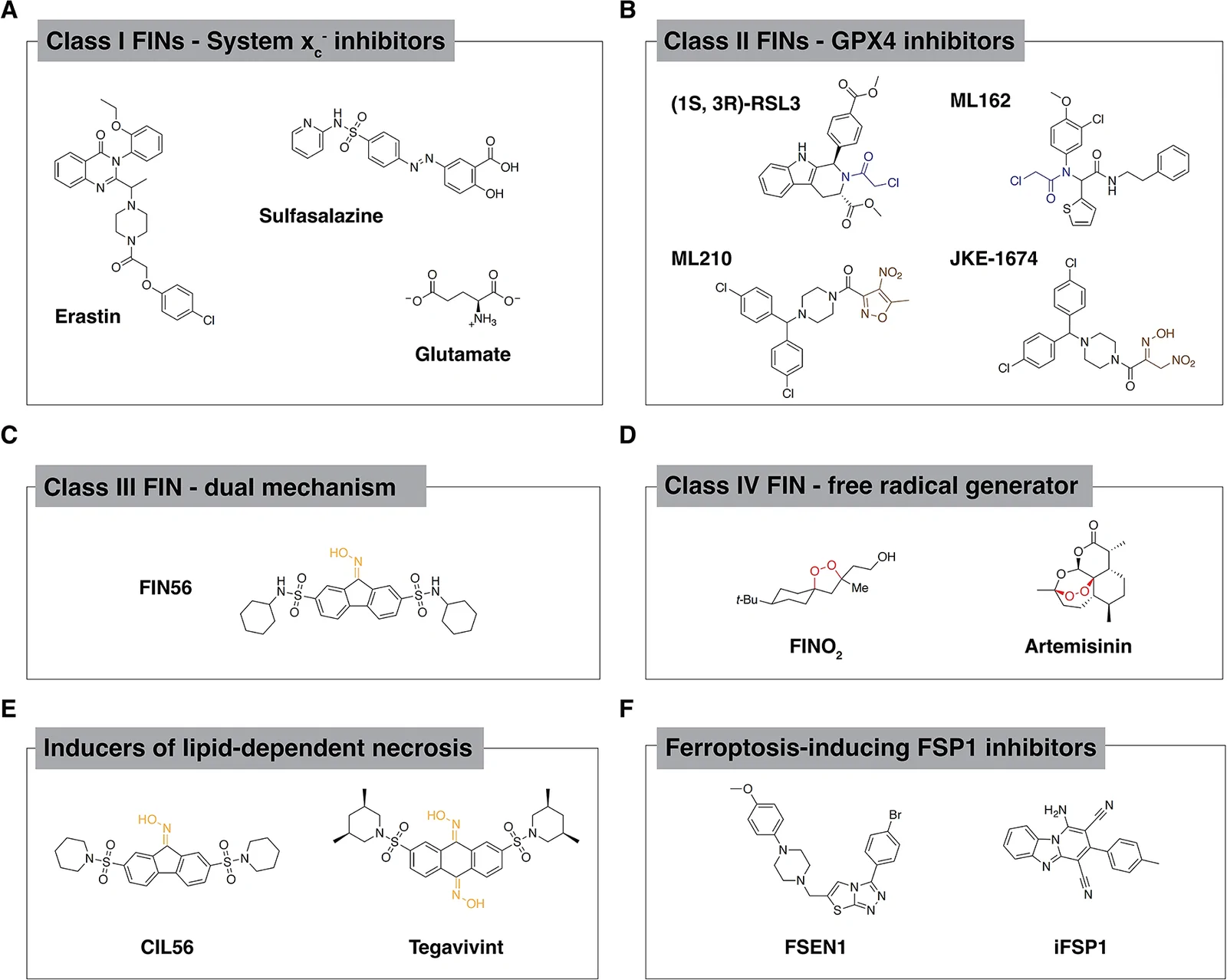
Structures of lethal molecules. (A) Structures of representative class I FINs; (B) Structures of representative class II FINs. Blue structural motifs indicate electrophilic warhead essential to covalent inhibition mechanism and brown structural motifs indicate the prodrug warhead that is converted to a reactive electrophile in cells; (C) Structure of prototypic oxime-containing class III FIN, FIN56 (oxime functional group drawn in yellow); (D) Structure of representative class IV FINs. The iron-reactive, radical-generating endoperoxide functional group is highlighted in red; (E) Structures of oxime-containing lethal compounds that are structurally related to the FIN56. The oxime motif, drawn in yellow, is essential for activation of lipid-dependent necrosis by these compounds; (F) Structures of representative FSP1 inhibitors, a more recently introduced class of ferroptosis inducers with distinct mechanism compared to previously defined classes. FINs: ferroptosis-inducing compounds; FSP1: ferroptosis suppressor protein 1; GPX4: glutathione peroxidase 4.

**Figure 2.**
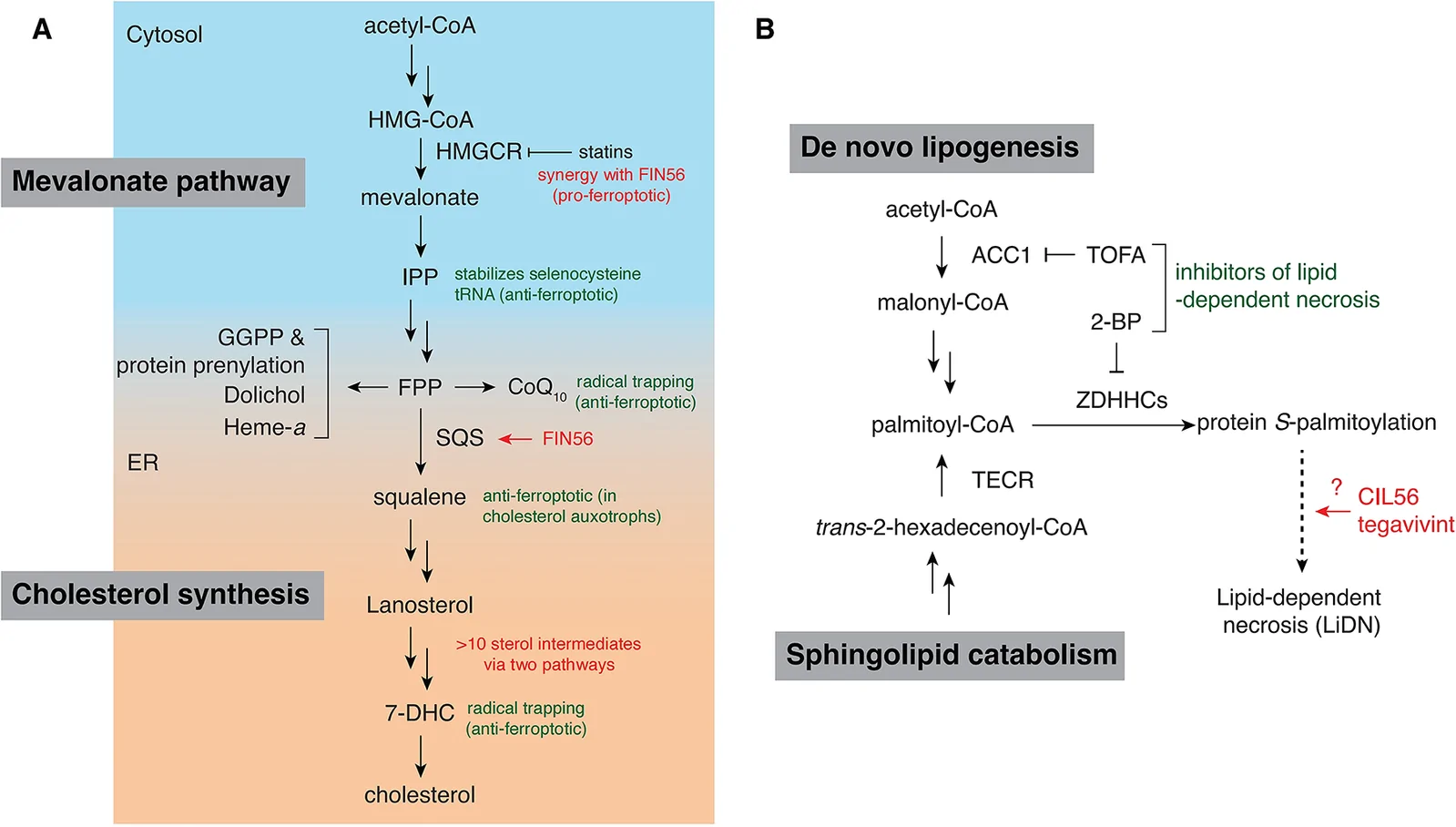
Proposed mechanisms of oxime-containing compound lethality. (A) A visual map of enzymes and metabolites in the mevalonate and cholesterol synthesis pathways that have been implicated in ferroptosis; (B) A simplified map of core lipid metabolic components essential to the execution of lipid-dependent necrosis induced by the oxime-containing lethal compounds CIL56 and tegavivint. CoA: coenzyme A; HMG-CoA: 3-hydroxy-3-methylglutaryl-coenzyme A; IPP: isopentenyl pyrophosphate; HMGCR: 3-hydroxy-3-methylglutaryl-CoA reductase; FPP:farnesyl pyrophosphate; GGPP: geranylgeranyl diphosphate; CoQ_10_: coenzyme Q10; SQS: squalene synthase; ER: endoplasmic reticulum; 7-DHC: 7-dehydrocholesterol; ACC1: acetyl-CoA carboxylase 1; TOFA: 5-(tetradeclyoxy)-2-furoic acid; 2-BP: 2-bromopalmitate; ZDHHCs: zinc finger DHHC domain-containing palmitoyl S-acyltransferases; TECR: trans-2,3-enoyl-CoA reductase.

**Table 1.** Comparison of chemical structures and mechanisms.

Compound	Core scaffold	Oxime number	Key inhibitors	Primary cell death modality	Genetic suppressors	Clinical status

FIN56	fluorenone	1	Fer-1, ATOC, TOFA	Ferroptosis	Unknown	N/A
CIL56	fluorenone	1	TOFA, 2-BP	LiDN	*ZDHHC5, TECR*	N/A
Tegavivint	anthraquinone	2	TOFA, 2-BP	LiDN	*TECR*	NCT07463599 (colorectal cancer) NCT07144254 (osteosarcoma) NCT03459469 (desmoid tumor) NCT04874480 (leukemia) NCT05797805 (hepatocellular carcinoma) NCT04851119 (multiple cancers) NCT05755087 (lymphoma) NCT04780568 (non-small cell lung cancer)

Fer-1: ferrostatin 1; ATOC: *α*-tocopherol; TOFA: 5-(tetradeclyoxy)-2-furoic acid; 2-BP: 2-bromopalmitate; ZDHHC5: zDHHC palmitoyltransferase 5; TECR: trans-2,3-enoyl-CoA reductase.

## Data Availability

Not applicable.
